# Molecular Diagnosis of Steroid 21-Hydroxylase Deficiency: A Practical Approach

**DOI:** 10.3389/fendo.2022.834549

**Published:** 2022-03-29

**Authors:** María Arriba, Begoña Ezquieta

**Affiliations:** ^1^ Molecular Diagnostics Laboratory, Department of Laboratory Medicine, Hospital General Universitario Gregorio Marañón, Madrid, Spain; ^2^ Gregorio Marañón Health Research Institute (IiSGM), Madrid, Spain

**Keywords:** ccongenital adrenal hyperplasia (CAH), 21-hydroxylase deficiency, *CYP21A2* gene, classical forms of congenital adrenal hyperplasia, non-classical forms of congenital adrenal hyperplasia, molecular diagnosis

## Abstract

Adrenal insufficiency in paediatric patients is mostly due to congenital adrenal hyperplasia (CAH), a severe monogenic disease caused by steroid 21-hydroxylase deficiency (21-OHD, encoded by the *CYP21A2* gene) in 95% of cases. *CYP21A2* genotyping requires careful analyses that guaranty gene-specific PCR, accurate definition of pseudogene-gene chimeras, gene duplications and allele dropout avoidance. A small panel of well-established disease-causing alterations enables a high diagnostic yield in confirming/discarding the disorder not only in symptomatic patients but also in those asymptomatic with borderline/positive results of 17-hydroxyprogesterone. Unfortunately, the complexity of this locus makes it today reluctant to high throughput techniques of massive sequencing. The strong relationship existing between the molecular alterations and the degree of enzymatic deficiency has allowed genetic studies to demonstrate its usefulness in predicting/classifying the clinical form of the disease. Other aspects of interest regarding molecular studies include its independence of physiological variations and analytical interferences, its usefulness in the diagnosis of simple virilizing forms in males and its inherent contribution to the genetic counseling, an aspect of great importance taking into account the high carrier frequency of CAH in the general population. Genetic testing of *CYP21A2* constitutes an irreplaceable tool to detect severe alleles not just in family members of classical forms but also in mild late-onset forms of the disease and couples. It is also helpful in areas such as assisted reproduction and preimplantation diagnosis. Molecular diagnosis of 21-OHD under expert knowledge definitely contributes to a better management of the disease in every step of the clinical course.

## 1 Introduction

Congenital adrenal hyperplasia (CAH) due to 21-hydroxylase deficiency (21-OHD)(OMIM #201910) is an inherited autosomal recessive disorder responsible of 95% of CAH cases (1, 2). It has its origin in a defect of steroid 21-hydroxylase (21-OH), an enzyme encoded by the *CYP21A2* gene. Alterations in *CYP21A2* cause an impairment of the enzymatic activity and leads to the accumulation of 17-hydroxyprogesterone (17-OHP), which is diverted towards formation of androgens (1, 3). As an actionable, non-infrequent and life-threatening disease, CAH is included in the neonatal screening of several countries (4).

Although 17-OHP is the metabolic marker of the deficiency, *CYP21A2* genotyping contributes as a diagnostic tool due to its independence on physiology and its strong relationship with clinical severity (4). The high carrier frequency (5, 6) and the recurrent impaired fertility in patients (7–9) further evidence the important contribution that genotyping does. Molecular studies provide valuable information in prevention and contribute to a better management of the disease (10, 11).

CAH is related to a wide range of clinical behaviors, with phenotypes varying from severe classical forms (CLF) to moderate late-onset non-classical forms (NCF). As a highly penetrant monogenic disease, 21-OHD shows a strong, although not complete, genotype-phenotype relationship in which the clinical features correspond to the less severely impaired allele (1, 12, 13). Variants causing null or minimal enzymatic activity in both alleles result in salt-wasting forms (SW), whereas their compound heterozygosity with variants causing residual activity result in simply virilizing forms (SV). NCF are due to mild alterations in homozygosity or a compound heterozygosity of either two mild alterations or a severe and a mild one (1, 3, 12, 14–16) (**Supplementary Tables 1**, **2**). Some lacks of genotype-phenotype relationship may result from extraadrenal 21-hydroxylation mediated by liver P450 cytochromes (17).

## 2 Gene Locus Structure and Nature of *CYP21A2* Alterations

*CYP21A2* is arranged in tandem with its inactive pseudogene (*CYP21A2P*) within a genetic unit designated as RCCX module, where also the genes *TNXA/B*, *C4A/B* and *RP* are harbored (18). Most chromosomes have two RCCX modules, although mono-, tri- or even quadrimodular arrangements have been described (19, 20). The high homology existing between gene and pseudogene (98% in coding and 96% in non-coding regions) together with that existing between RCCX modules favor unequal cross-overs during meiosis making that most pathological alleles in CAH arise from mechanisms of asymmetric recombination (25-30%) and gene conversion events (70%). Consequently, *CYP21A2* genotyping requires careful analyses that guaranty gene-specific PCR with allele dropout avoidance, and accurate definition of pseudogene-gene chimeras and gene duplications. Of course, an expert interpretation of the results is needed.

### 2.1 Alterations Due to Intrinsic Locus-Derived Mechanisms

#### 2.1.1 Point Pathological Variants: Microconversions

**Figure 1:**
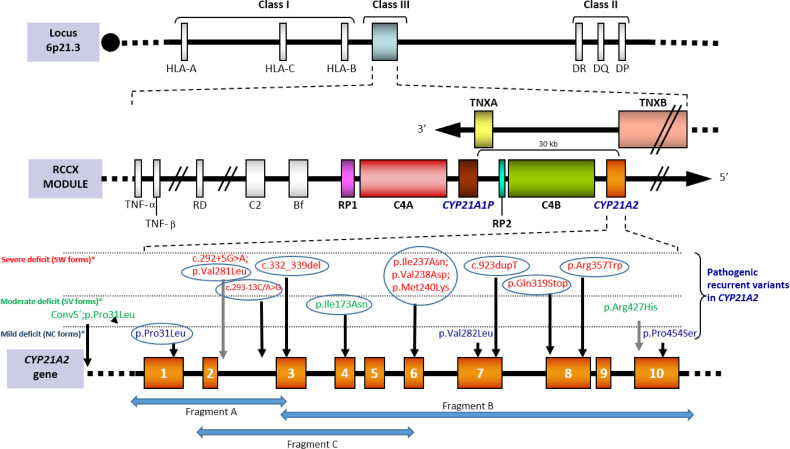
[Adapted from Santomé et al., (21)]. Scheme of the RCCX module located on the short arm of chromosome 6 within the HLA class III region. Tandem duplication affects *CYP21* and C4 genes. In humans only *CYP21A2* gives rise to the functional protein, whereas *CYP21P* is a homologous pseudogene that includes several inactivating point variants that can be transferred to the active gene by small gene conversion events. Both C4A and C4B are functional. Tenascin, also duplicated, is encoded in the complementary chain. The bottom of the image shows the recurrent variants grouped according to how they affect the enzimatic functionality: severely (red), moderately severe (green) or mildly (blue). Recurrent variants in all populations are circled. The complete nomenclature of each variant including the cDNA position (NM_000500.9) would be: c.92C>T [p.Pro31Leu], c.292+5C>A, c.293-13C>G, c.332-339del, c.518T>A [p.Ile173Asn], c.(710T>A; 713T>A; 719T>A) p.[Ile237Asn; Val238Glu; Met240Lys], c.844G>T [p.Val282Leu], c.923dupT, c.955C>T [p.Gln319*], c.1069C>T [p.Arg357Trp], c.1280G>A [p.Arg427His] and c.1360C>T [p.Pro454Ser]). The arrows “Fragment A”, “Fragment B” and “Fragment C” represents specific amplicons for *CYP21A2* amplification.

Around 70% of the disease causing alterations in CAH are pseudogene-deleterious-variants that have been transferred to the gene by small gene conversion events. As a result, a limited group of pathogenic variants with well-known phenotypic effects is present in all populations (3) (**Figure 1**).

#### 2.1.2 Gene Chimeras

Asymmetric recombination between *CYP21A2* and *CYP21A2P* is responsible of about 25-30% of all deficient alleles (3, 22, 23). This mechanism results in the appearance of pseudogene-gene chimeras (traditionally named “gene deletions”) usually extending from somewhere between exons 3 and 8 of *CYP21A2P* to the corresponding point in *CYP21A2*, yielding a non-functional gene in which the 5’-end corresponds to *CYP21A2P* and the 3’-end corresponds to *CYP21A2*. It is important to mention a subset of patients in which the deletion is extended into the *TNXB* gene resulting in a contiguous gene syndrome named CAH-X consisting in CAH and Ehlers-Danlos Syndrome (24) that should also be investigated (25).

Chimeras are usually categorized into classic and attenuated depending on the location of the junction site, having been reported nine different types (26). Classic types contain the c.293-13C>G region and produce non-functional alleles whereas attenuated ones have the junction upstream of that region and associate a less severe phenotype (26) (see *Avoidable Pitfalls Upon Complementary Characterization of Alleles*).

#### 2.1.3 *De Novo* Alterations

*De novo* alterations (1-2% of all 21OH-deficient alleles) are usually derived from gene recombination processes (27–30), being therefore detectable in the basic screening of recurrent variants.

### 2.2 Alterations Due to Conventional Mechanisms

Alterations other than those derived from recombinant events are less frequent and usually involve functional residues, generate frameshifts or stop codons (16, 21, 30, 31). The number of splicing pathological variants described so far is small (30), with a new candidate recently reported (32). Alterations in regulatory regions are controversial and difficult to demonstrate but tend to be mild changes. To date, more than 200 different pathogenic variants in *CYP21A2* have been described (16, 30, 31).

## 3 Disease Frequency and Origin of *CYP21A2* Alterations

CAH constitutes a non-infrequent disease, even in its severe neonatal forms. This seems to be the result of the prolific molecular mechanisms previously mentioned, although a founder effect has also been proposed (33–37). Regarding this latter, some studies have documented a lower mortality in *CYP21A2* carriers mainly due to a decreased number of infections in these individuals (38).

Considering that *de novo* variants in *CYP21A2* are infrequent (27, 29, 30) and that alterations are maintained through generations once originated (33, 39), it is not uncommon that the presence of new/rare pathological variants be the result of the dissemination of single original alleles.

## 4 Genetic Diagnosis of CAH

Since CAH due to 21-OHD accounts for 95% of all CAH cases, *CYP21A2* should be the first gene to investigate in males and virilized girls with adrenal insufficiency. The remaining genes causing CAH (1, 2, 12, 40) as well as other involved in adrenal insufficiency (41) should be investigated using high-throughput approaches (massive sequencing gene panels) (40–42). On regard *CYP11B1*, it is important to highlight its high homology with *CYP11B2* and the consequent existence of hybrid genes (43–45).

## 5 *CYP21A2* Genotyping

Traditional approaches for *CYP21A2* genotyping usually include methods such as capillary sequencing, allele-specific oligonucleotide hybridization, SNaPshot minisequencing and MLPA, which are labor intensive and have limited multiplexing capability, but which keep being used given their proven clinical usefulness and the difficulty of optimizing the current massive sequencing technologies to this complex locus. Conventional massive platforms are poorly equipped to characterize gene-pseudogene pairs and have the limitation of being based on PCR-amplifications and uniquely aligning short reads (that may not include *CYP21A2* gene-specific regions). As a consequence, they are not still the first-choice option for *CYP21A2* genotyping although some promising results have been obtained (31, 46–49). Third-generation platforms based on direct sequencing of long DNA strands without previous amplification seem promising tools (50–52).

### 5.1 Detection of Point Pathological Variants: Gene-Specific PCR

Current strategies for the specific amplification of *CYP21A2* rely on regions that are known to be different from those of the pseudogene, either as targets for restriction sites prior to PCR or PCR-specific primers. Since one of these latter regions is located on exon 3 (where the variant c.332-339del is located in *CYP21A2P*), an extensively used scheme for the specific amplification of *CYP21A2* is obtaining two fragments (one from 5’UTR to exon 3 and another from exon 3 to 3'UTR). However, alleles carrying the variant c.332-339del as a single microconversion would not be detected in this way (neither chimeras, conversions or gene duplications including it), so a third fragment in which the 3’-end is located on the specific site on exon 6 (where the cluster of three variants is harbored in *CYP21A2P*) can be incorporated (34, 39, 53–56) (**Figure 1**). This last overlapping fragment allows the PCR-detection of pseudogene-gene chimeras with the breaking point before exon 6.

Recurrent variants (**Figure 1**) may be investigated in a first screening performed by allele-specific oligonucleotide hybridization or SNaPshot minisequencing, although they and other point variants are detected with Sanger sequencing on these gene-specific fragments. Whole gene sequencing must guarantee an accurate interpretation based on well-documented alterations due to the lack of complete knowledge regarding the impact of every variant in this small but polymorphic gene. *In vitro* analyses (57–59) and/or models investigation (60–62) should support the involvement of new variants, but only clinical validation in different populations and genotypes will confirm their causality.

Segregation of alterations in parental samples is an important issue since gene chimeras and large or double micro-conversions include several alterations within the same allele (carrier status), a very different situation from that in which alterations are located in different alleles (affected patient). Approximately 5-7% of affected alleles carrying two or more point alterations (63). Patients carrying gene chimeras/conversions that include the specific regions used in PCR protocols result in hemizygosity and directly stablish the segregation, although not the carrier status of progenitors (see *Family Studies*).

### 5.2 Analysis of Gene Chimeras: MLPA

MLPA allows to identify gene deletions/conversions avoiding the inconvenients linked to Southern blotting. Nevertheless, since it also has unavoidable limitations [reduction of signal when alterations/polymorphisms exist in a probe-binding region (64), inability to detect the *cis*/*trans* disposition of the alterations, or lack of probes addressed to some frequent variants], must be always complemented with other analyses. Unfortunately, a comprehensive revision defining every MLPA pattern and its deduced genotype is still lacking, although some studies are contributing to a better definition of this issue (65). MLPA should also be applied in the complementary characterization of some complex alleles (see *Avoidable Pitfalls Upon Complementary Characterization of Alleles*).

### 5.3 Avoidable Pitfalls Upon Complementary Characterization of Alleles

Some of the seemingly lacks of genotype-phenotype relationship in several frequent point variants are not further sustained when alleles are better characterized. An efficient multistep approach (64) allows a comprehensive mutation analysis. Apparently mild alleles which are not really such are those carrying the variant c.92C>T [p.Pro31Leu] with a *cis* pseudogene-conversion in 5’ (26, 56, 66, 67), and those carrying the variant c.844G>T [p.Val282Leu] in *cis* with the intronic change c.292+5G>A, an alteration observed in SW from Mediterranean populations (15, 68) (**Figure 1** and **Supplementary Table 1**).

Examples of “severe” alleles that are not really such are those with the variant c.955C>T [p.Gln319*] and two copies of the gene, present in several populations (6, 30, 69) (**Supplementary Table 1**). Fortunately, since the whole gene is involved in these alleles, MLPA allows its detection in spite of the salsa MLPA Probemix P050-C1 CAH (MRC Holland) no longer includes exon 8 probes. It is important to mention that some of these alleles carry additional alterations [e.g. c.518T>A (p.Ile173Asn) or the combination of c.293-13 C>G and c.332_339del (6, 70)] and are severe. Some gene conversions involving exons 4 to 8 are not such deficient-alleles. Pseudogenes including the gene-specific region in exon 3, although infrequent (71), result in an identical pattern upon PCR amplification, so these conversions should be investigated with a complementary MLPA analysis.

Discrimination of homo/hemizygosity of mild variants is crucial in NCF (72) as hemizygosity requires genetic counseling. Also essential is to guarantee the efficient amplification of both alleles in order to avoid incorrect interpretations such as false homozygotes due to allele dropout of the normal allele (73). A complementary indirect analysis also provides useful information preventing serious mistakes in prenatal samples (see *Usefulness of an Indirect Analysis*).

## 6 Usefulness of an Indirect Analysis

Indirect analyses performed by either microsatellite typing or SNPs (6, 74–76) are a useful tool (see *Contribution of CYP21A2 Genotyping*) since informative polymorphic markers on both sides of the gene in each family configure distinct haplotypes in normal and affected chromosomes. They are helpful with prenatal samples, in preimplantation studies and in allele segregation, being able to reveal/discard consanguinity in patients carrying rare variants in homozygosity [useful as a complement of a basic/first study in patients with borderline/false positive results in the neonatal screening (see *Neonatal Screening*)]. Also in epidemiology, since the same haplotype for a new variant in unrelated patients suggests the variant dissemination and the potential interest of its inclusion in the basic screening of that population. Some informative microsatellite loci flanking the *CYP21A2* gene are D6S2792-D6S273 and D6S1014- D6S439 together with two intronic ones in genes *TNF* and *TAP1* (34, 63, 74, 77, 78).

## 7 Contribution of *CYP21A2* Genotyping

### 7.1 Neonatal Clinical Suspicion

Although clinical manifestations such as adrenal insufficiency or virilization in girls perform the suspicion in the neonatal period, there are unspecific signs (e.g. hypoglycemia, clitoromegaly or genital hyperpigmentation) frequent in combination with 17-OHP elevations (4, 79). Genotyping of *CYP21A2* allows to confirm/discard the disease in both scenarios (80) especially when analytical interferences in the direct immunoassay exist (81, 82). Comprehensive *CYP21A2* genotyping should be guaranteed paying special attention to variants with a significance still poorly established. Failure to detect well-stablished pathogenic variants in *CYP21A2* must prompt further studies.

### 7.2 Neonatal Screening

Clinical guidelines recommend a second-tier analysis by liquid chromatography–tandem mass spectrometry to improve the positive predictive value of CAH screening (4). Neonates with borderline/high levels of 17-OHP in these programs can take benefit from *CYP21A2* analyses (80, 83–86). Not only CLF, but also neonatal cryptic forms (NCF and SV in males) are detected at this stage, being molecular studies able to correctly classify them (11, 80, 86, 87) by a firstly analysis focused on the identification of recurrent variants (in order to eliminate uncertainty) followed by Sanger sequencing when just one deficient-allele or microsatellite-homozygosity is detected (80).

### 7.3 Non-Classical Forms

The high recurrence of c.844G>T [p.Val282Leu] in some populations (88, 89) helps to “unmask” severe alleles through the clinical expressiveness of NCF [70% carrying severe alleles (4); 41% in paediatric patients, **Supplementary Table 1**]. *CYP21A2* should be always considered in NCF to allow a proper genetic counseling.

**Figure 2:**
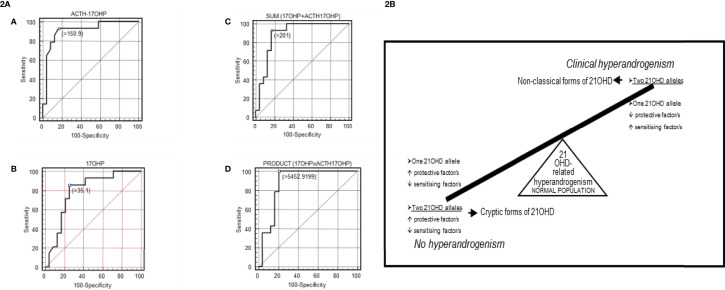
**(2A)** [Taken from Ezquieta et al., (56)]: Receiver operating characteristic (ROC) curves analyses in fully genotyped children affected with NCF of 21-OHD (mild/mild *vs* severe/mild genotype) for: **(A)** adrenocorticotropic hormone (ACTH)-stimulated 17-OHP, **(B)** basal 17-OHP, or **(C, D)** the combination of both parameters [**(C)**: sum; **(D)**: product]. Areas under the curves (SE): ACTH-stimulated 17-OHP, 0.908 (0.057); basal 17-OHP, 0.790 (0.081); sum 0.866 (0.068); product 0.884 (0.064). Cut-off values, nmol/L **(A–C)** and nmol^2^/L^2^ **(D)**. The cut-offs for maximum predictive values are represented by small, empty squares in the Figures. **(2B)** [From Ezquieta et al., (91)] Diagram of a hypothetical interaction between protective and sensitizing factors modulating the clinical expressivity of 21-OHD-related hyperandrogenism. CAPN10-UCSNP44C and TNFR2-R196 are proposed in this study to be sensitizing and protective factors, respectively.

Levels of 17-OHP, either basal or post-ACTH, constitute the most sensitive parameter to define a NCF since *CYP21A2* mild alterations are not fully characterized. A proper threshold for 17-OHP values is difficult to define since some carriers are prone to present a hyperandrogenism similar to that shown in NCF (56, 85, 90–92). Genotyped carriers inside fully characterized segregated families are useful to achieve this goal (56, 91). Compound heterozygosity with severe alleles in NCF may be suspected based on 17-OHP levels (**Figure 2A** and **Supplementary Table 3**) (56, 63) conversely to what happen with carriers of severe *vs.* mild variants.

Monogenic and polygenic models in paediatric hyperandrogenism due to 21-OHD have been detected (91)(**Figure 2B**), being carriers (monoallelic) with hyperandrogenism the counterpart of cryptic forms (biallelic alterations) without clinical expression. Considering the important contribution of the “back door” pathway to circulating levels of the potent androgen 11-ketotestosterone in CAH (93, 94), investigation of gene variants coding for the enzymes involved seems interesting.

### 7.4 Carrier Detection

The biochemical marker 21-deoxicortisol detects carriers (10, 40, 95), although only molecular analyses are able to discriminate carriers of severe alleles. Individuals with hyperandrogenism and moderately elevated post-ACTH 17-OHP levels (not reaching the NCF threshold) may take benefit from *CYP21A2* analyses since alterations are more frequent in these patients (91) (**Supplementary Table 2**).

### 7.5 Genetic Counseling

The high carrier frequency of severe variants in general population (about 1:60) (15, 96, 97) (**Supplementary Table 1**, false severe alleles) makes reasonable to refine the risk of having an affected child by genotyping *CYP21A2* in couples where one member is affected/carrier. Individuals with CLF present a risk of 1:120 of having a newborn affected with a CLF. The theoretical risk is lower in NCF [1:250 (4)] although some studies have documented to be higher (1.5-2.5%) (98).

### 7.6 Family Studies

Family studies are necessary to ascertain parental genotype and segregation of the pathological alleles among the offspring. They are initially addressed to detect/discard alterations documented in the index case, but the high carrier frequency justifies the subsequent screening of frequent pathological variants to discard its coexistence in the family. Progenitors must not be considered obligate carriers since *de novo* variants are detected in 1-2% of deficient alleles (27, 29, 30).

### 7.7 Assisted Reproductive Techniques and Genetic Counseling

CAH due to 21-OHD should be considered in reproductive assistance and genetic counseling (7, 99, 100) due to the associated infertility (7–9, 40) and the high frequency of carriers in general population. The strong genotype-phenotype relationship (13, 101) facilitates counseling in couples even in absence of an index case, but it should not be forgotten that expressivity vary particularly in moderately-severe forms (13).

### 7.8 Prenatal Diagnosis

Prenatal studies are normally performed inside CLF-families. It is still accomplished on samples from corionic villus through direct analysis addressed to investigate those alterations detected in the index case. An additional indirect analysis (6, 15, 74) provides the possibility of detecting maternal contamination and avoids eventual allele dropout artefacts.

Prenatal treatment prevents virilization in girls affected with CAH but is still considered experimental (4). Prenatal diagnosis establishes treatment withdraw in non-affected foetus (carriers and non-carriers). Protocols must include screening for Y-chromosomal DNA in maternal blood (4) to minimize (40) treatment in males. Since prenatal treatment is only effective if established at 6^th^-7^th^ weeks (4, 102), it is unfeasible totally avoid treatment in males since cfDNA analyses must be performed in samples with a foetal fraction about 3.5-4% (9^th^-10^th^ week).

*CYP21A2* genotyping from cfDNA in maternal blood is a promising approach not suitable in clinical settings yet (4, 102). For its application, massive sequencing based on an indirect analyses conducted by SNP-haplotypes previously defined in parents and index case is necessary due to the coexistence of foetal and maternal DNA in the same sample (76).

### 7.9 Preimplantation Genetic Diagnosis

This particular approach enables to study the embryo before the transference to the uterus. These tests are mentioned in the last Clinical Practice Guidelines from the Endocrine Society although subjected to their own risk and ethical controversies (4).

Microsatellite typing is the most appropriate approach, since the paucity of sample hampers a direct gene analysis and haplotypes detected in the directly genotyped index case provide the information.

### 7.10 Other Prenatal Scenarios

Suspicion of CAH due to anomalies detected by fetal ultrasound or genetic counseling for a couple at risk (not previously genotyped) with an ongoing pregnancy are prenatal situations in which *CYP21A2* genotyping are requested. When the index case is unknown, the only suitable approach is the direct analysis. Only well-documented pathogenic alterations should be considered.

## 8 Conclusions

*CYP21A2* genotyping favorably contributes to confirm/discard CLF after neonatal or prenatal suspicion. The high frequency of carriers in general population and the infertility associated to the disease turn molecular diagnosis into an irreplaceable tool to detect/discriminate severe alleles in family members and in genetic counseling, as well as in specific areas as assisted reproduction and preimplantational diagnosis. The high complexity of the locus makes essential the performance of *CYP21A2* genotyping under supervision of expert personnel in the field. There is no doubt that molecular diagnosis of 21-OHD definitely contributes to a better management of the disease in every step of the clinical course.

## Author Contributions

Conception and design: MA and BE. Manuscript writing: MA and BE. Manuscript revision: MA and BE. All authors contributed to the article and approved the submitted version.

## Conflict of Interest

The authors declare that the research was conducted in the absence of any commercial or financial relationships that could be construed as a potential conflict of interest.

## Publisher’s Note

All claims expressed in this article are solely those of the authors and do not necessarily represent those of their affiliated organizations, or those of the publisher, the editors and the reviewers. Any product that may be evaluated in this article, or claim that may be made by its manufacturer, is not guaranteed or endorsed by the publisher.

## References

[1] El-Maouche D Arlt W Merke DP. Congenital Adrenal Hyperplasia. Lancet (2017) 390:2194–210. doi: 10.1016/S0140-6736(17)31431-9 28576284

[2] Miller WL. Mechanisms in Endocrinology: Rare Defects in Adrenal Steroidogenesis. Rev Eur J Endocrinol (2018) 179(3):R125–41. doi: 10.1530/EJE-18-0279 29880708

[3] White PC Speiser PW. Congenital Adrenal Hyperplasia Due to 21-Hydroxylase Deficiency. Endocr Rev (2000) 21:245–91. doi: 10.1210/edrv.21.3.0398 10857554

[4] Speiser PW Arlt W Auchus RJ Baskin LS Conway GS Merke DP. Congenital Adrenal Hyperplasia Due to Steroid 21-Hydroxylase Deficiency: An Endocrine Society Clinical Practice Guideline. J Clin Endocrinol Metab (2018) 103:4043–88. doi: 10.1210/jc.2018-01865 PMC6456929 30272171

[5] Baumgartner-Parzer SM Nowotny P Heinze G Waldhäusl W Vierhapper H. Carrier Frequency of Congenital Adrenal Hyperplasia (21-Hydroxylase Deficiency) in a Middle European Population. J Clin Endocrinol Metab (2005) 90:775–8. doi: 10.1210/jc.2004-1728 15572419

[6] Ezquieta B Beneyto M Muñoz-Pacheco R Barrio R Oyarzabal M Lechuga JL. Gene Duplications in 21-Hydroxylase Deficiency: The Importance of Accurate Molecular Diagnosis in Carrier Detection and Prenatal Diagnosis. Prenat Diagn (2006) 26(12):1172–8. doi: 10.1002/pd.1584 17042033

[7] Ezquieta B Alonso M Álvarez E Arnao DR Rodríguez A Siguero JP. Should 21-Hydroxylase Deficiency Genotyping be Considered in Assisted Reproductive Technology Programs? Fertil Steril (2007) 88(5):1437.e5–11. doi: 10.1016/j.fertnstert.2007.01.030 17481616

[8] Reichman DE White PC New MI Rosenwaks Z. Fertility in Patients With Congenital Adrenal Hyperplasia. Fertil Steril (2014) 101(2):301–9. doi: 10.1016/j.fertnstert.2013.11.002. Fertility in patients with congenital adrenal hyperplasia. 24355046

[9] Carmina E Dewailly D Escobar-Morreale HF Kelestimur F Moran C Oberfield S. Non-Classic Congenital Adrenal Hyperplasia Due to 21-Hydroxylase Deficiency Revisited: An Update With a Special Focus on Adolescent and Adult Women. Hum Reprod Update (2017) 23(5):580–99. doi: 10.1093/humupd/dmx014 28582566

[10] Forest MG Tardy V Nicolino M David M Morel Y. 21-Hydroxylase Deficiency: An Exemplary Model of the Contribution of Molecular Biology in the Understanding and Management of the Disease. Ann Endocrinol (Paris) (2005) 66(3):225–32. doi: 10.1016/s0003-4266(05)81754-8 15988383

[11] Lajic S Karlsson L Zetterström RH Falhammar H Nordenström A. The Success of a Screening Program Is Largely Dependent on Close Collaboration Between the Laboratory and the Clinical Follow-Up of the Patients. Int J Neonatal Screen (2020) 6(3):68. doi: 10.3390/ijns6030068 33117907 PMC7569867

[12] Krone N Arlt W. Genetics of Congenital Adrenal Hyperplasia. Best Pract Res Clin Endocrinol Metab (2009) 23(2):181–92. doi: 10.1016/j.beem.2008.10.014 PMC5576025 19500762

[13] New MI Abraham M Gonzalez B Dumic M Razzaghy-Azar M Chitayat D. Genotype-Phenotype Correlation in 1,507 Families With Congenital Adrenal Hyperplasia Owing to 21-Hydroxylase Deficiency. Proc Natl Acad Sci USA (2013) 110(7):2611–6. doi: 10.1073/pnas.1300057110 PMC3574953 23359698

[14] Speiser PW Azziz R Baskin LS Ghizzoni L Hensle TW Merke DP. Congenital Adrenal Hyperplasia Due to Steroid 21-Hydroxylase Deficiency: An Endocrine Society Clinical Practice Guideline. J Clin Endocrinol Metab (2010) 95:4133–60. doi: 10.1210/jc.2009-2631 PMC2936060 20823466

[15] Ezquieta B Santomé L Barrio R Barrionuevo JL López-Siguero JP Oliver A. Carrier Detection and Prenatal Diagnosis of Congenital Adrenal Hyperplasia Must Identify 'Apparently Mild' CYP21A2 Alleles Which Associate Neonatal Salt-Wasting Disease. Prenat Diagn (2010) 30(8):758–63. doi: 10.1002/pd.2537 20661889

[16] Concolino P Costella A. Congenital Adrenal Hyperplasia (CAH) Due to 21-Hydroxylase Deficiency: A Comprehensive Focus on 233 Pathogenic Variants of CYP21A2 Gene. Rev Mol Diagn Ther (2018) 22(3):261–80. doi: 10.1007/s40291-018-0319-y 29450859

[17] Gomes LG Huang N Agrawal V Mendonça BB Bachega TA Miller WL. Extraadrenal 21-Hydroxylation by CYP2C19 and CYP3A4: Effect on 21-Hydroxylase Deficiency. J Clin Endocrinol Metab (2009) 94(1):89–95. doi: 10.1210/jc.2008-1174 18957504 PMC2630875

[18] Yang Z Mendoza AR Welch TR Zipf WB Yu CY. Modular Variations of HLA Class III Genes for Serine/Threonine Kinase RP, Complement C4, Steroid 21-Hydroxylase CYP21 and Tenascin TNX (RCCX). A Mechanism for Gene Deletions and Disease Associations. J Biol Chem (1999) 274:12147–56. doi: 10.1074/jbc.274.17.12147 10207042

[19] Chung EK Yang Y Rennebohm RM Lokki ML Higgins GC Jones KN. Genetic Sophistication of Human Complement Components C4A and C4B and RP-C4-CYP21-TNX (RCCX) Modules in the Major Histocompatibility Complex. Am J Hum Genet (2002) 71(4):823–37. doi: 10.1086/342777 PMC378539 12226794

[20] Sweeten TL Odell DW Odell JD Torres AR. C4B Null Alleles are Not Associated With Genetic Polymorphisms in the Adjacent Gene CYP21A2 in Autism. BMC Med Genet (2008) 9:1. doi: 10.1186/1471-2350-9-1 18179706 PMC2265260

[21] Santomé JL Cirujano A Ferreiro B Casado C Muñoz-Pacheco R Ezquieta B. Simple Virilizing Forms of Congenital Adrenal Hyperplasia: Adaptation and Prospective Validation of the Molecular Screening [Article in Spanish]. Med Clin (Barc) (2010) 135(5):195–201. doi: 10.1016/j.medcli.2009.11.039 20171703

[22] Concolino P Mello E Minucci A Giardina E Zuppi C Toscano V. A New CYP21A1P/CYP21A2 Chimeric Gene Identified in an Italian Woman Suffering From Classical Congenital Adrenal Hyperplasia Form. BMC Med Genet (2009) 10:72. doi: 10.1186/1471-2350-10-72 19624807 PMC2718876

[23] Vrzalová Z Hrubá Z Hrabincová ES Vrábelová S Votava F Koloušková S. Chimeric CYP21A1P/CYP21A2 Genes Identified in Czech Patients With Congenital Adrenal Hyperplasia. Eur J Med Genet (2011) 54(2):112–7. doi: 10.1016/j.ejmg.2010.10.005 20970527

[24] Miller WL Merke DP. Tenascin-X, Congenital Adrenal Hyperplasia, and the CAH-X Syndrome. Horm Res Paediatr (2018) 89(5):352–61. doi: 10.1159/000481911 PMC6057477 29734195

[25] Lao Q Merke DP. Molecular Genetic Testing of Congenital Adrenal Hyperplasia Due to 21-Hydroxylase Deficiency Should Include CAH-X Chimeras. Eur J Hum Genet (2021) 29(7):1047–8. doi: 10.1038/s41431-021-00870-5 PMC8298381 33824469

[26] Chen W Xu Z Sullivan A Finkielstain GP Van Ryzin C Merke DP. Junction Site Analysis of Chimeric CYP21A1P/CYP21A2 Genes in 21-Hydroxylase Deficiency. Clin Chem (2012) 58(2):421–30. doi: 10.1373/clinchem.2011.174037 PMC5576027 22156666

[27] Koppens PF Hoogenboezem T Degenhart HJ. Duplication of the CYP21A2 Gene Complicates Mutation Analysis of Steroid 21-Hydroxylase Deficiency: Characteristics of Three Unusual Haplotypes. Hum Genet (2002) 111(4-5):405–10. doi: 10.1007/s00439-002-0810-7 12384784

[28] Díez I Rodríguez A González E Martínez M Rodríguez B Ezquieta B. Virilizing Congenital Adrenogenital Syndrome With a *De Novo* I172N Mutation: Study of a New Case. Case Rep Pediatr (Barc) (2010) 72(1):72–8. doi: 10.1016/j.anpedi.2009.08.006 19819201

[29] Lopes da Silva-Grecco R de Paula D Rodrigues C Pontes P da Cunha HM Palandi-de-Mello M. A *De Novo* Mutation in CYP21A2 Gene in a Case of *In Vitro* Fertilization. Mol Genet Metab Rep (2015) 5:98–102. doi: 10.1016/j.ymgmr.2015.10.011 28649552 PMC5471403

[30] Baumgartner-Parzer S Witsch-Baumgartner M Hoeppner W. EMQN Best Practice Guidelines for Molecular Genetic Testing and Reporting of 21-Hydroxylase Deficiency. Eur J Hum Genet (2020) 28(10):1341–67. doi: 10.1038/s41431-020-0653-5 PMC7609334 32616876

[31] Simonetti L Bruque CD Fernández CS Benavides-Mori B Delea M Kolomenski JE. CYP21A2 Mutation Update: Comprehensive Analysis of Databases and Published Genetic Variants. Hum Mutat (2018) 39(1):5–22. doi: 10.1002/humu.23351 29035424

[32] Arriba M Oriola J Ezquieta B. A New Synonymous Variant Involving an mRNA Splicing Site in CYP21A2 Detected in 12 Unrelated Patients With Deficiency of 21-Hydroxylase. Clin Genet (2021) 100(5):634–6. doi: 10.1111/cge.14035 34370296

[33] Levo A Jääskeläinen J Sistonen P Sirén MK Voutilainen R Partanen J. Tracing Past Population Migrations: Genealogy of Steroid 21-Hydroxylase (CYP21) Gene Mutations in Finland. Eur J Hum Genet (1999) 7(2):188–96. doi: 10.1038/sj.ejhg.5200262 10196702

[34] Ezquieta B Cueva E Oyarzabal M Oliver A Varela JM Jariego C. Gene Conversion (655 Splicing Mutation) and the Founder Effect (Q318X) Contribute to the Most Frequent Severe Point Mutations in Congenital Adrenal Hyperplasia in the Spanish Population. Clin Genet (2002) 62:181–8. doi: 10.1034/j.1399-0004.2002.620213.x 12220458

[35] Billerbeck AE Mendonca BB Pinto EM Madureira G Arnhold IJ Bachega TA. Three Novel Mutations in CYP21 Gene in Brazilian Patients With the Classical Form of 21-Hydroxylase Deficiency Due to a Founder Effect. J Clin Endocrinol Metab (2002) 87(9):4314–7. doi: 10.1210/jc.2001-011939 12213891

[36] Kleinle S Lang R Fischer GF Vierhapper H Waldhauser F Födinger M. Duplications of the Functional CYP21A2 Gene Are Primarily Restricted to Q318X Alleles: Evidence for a Founder Effect. J Clin Endocrinol Metab (2009) 94(10):3954–8. doi: 10.1210/jc.2009-0487 19773403

[37] Silveira EL Elnecave RH dos Santos EP Moura V Pinto EM van der Linden I. Molecular Analysis of CYP21A2 Can Optimize the Follow-Up of Positive Results in Newborn Screening for Congenital Adrenal Hyperplasia. Clin Genet (2009) 76(6):503–10. doi: 10.1111/j.1399-0004.2009.01274.x 19930153

[38] Nordenström A Svensson J Lajic S Frisén L Nordenskjöld A Norrby C. Carriers of a Classic CYP21A2 Mutation Have Reduced Mortality: A Population-Based National Cohort Study. J Clin Endocrinol Metab (2019) 104:6148–54. doi: 10.1210/jc.2019-01199 31393570

[39] Ezquieta B Oyarzábal M Jariego CM Varela JM Chueca M. A Novel Frameshift in the first Exon of the 21-OH Gene Found Inhomozygosity in an Apparently Nonconsanguineous Family. Horm Res (1999) 51:135–41. doi: 10.1159/000023346 10461019

[40] Buonocore F Maharaj A Qamar Y Koehler K Suntharalingham JP Chan LF. Genetic Analysis of Pediatric Primary Adrenal Insufficiency of Unknown Etiology: 25 Years' Experience in the UK. J Endocr Soc (2021) 5(8):bvab086. doi: 10.1210/jendso/bvab086 34258490 PMC8266051

[41] Flück CE. MECHANISMS IN ENDOCRINOLOGY: Update on Pathogenesis of Primary Adrenal Insufficiency: Beyond Steroid Enzyme Deficiency and Autoimmune Adrenal Destruction. Eur J Endocrinol (2017) 177(3):R99–111. doi: 10.1530/EJE-17-0128 28450305

[42] Roucher-Boulez F Mallet-Motak D Tardy-Guidollet V Menassa R Goursaud C Plotton I. News About the Genetics of Congenital Primary Adrenal Insufficiency. Ann Endocrinol (Paris) (2018) 79(3):174–81. doi: 10.1016/j.ando.2018.03.016 29661472

[43] Hampf M Dao NT Hoan NT. Bernhardt R.Unequal Crossing-Over Between Aldosterone Synthase and 11beta-Hydroxylase Genes Causes Congenital Adrenal Hyperplasia. J Clin Endocrinol Metab (2001) 86(9):4445–52. doi: 10.1210/jcem.86.9.7820 11549691

[44] Portrat S Mulatero P Curnow KM Chaussain JL Morel Y Pascoe L. Deletion Hybrid Genes, Due to Unequal Crossing Over Between CYP11B1 (11beta-Hydroxylase) and CYP11B2(aldosterone Synthase) Cause Steroid 11beta-Hydroxylase Deficiency and Congenital Adrenal Hyperplasia. J Clin Endocrinol Metab (2001) 86(7):3197–201. doi: 10.1210/jcem.86.7.7671 11443188

[45] Ezquieta B Luzuriaga C. Neonatal Salt-Wasting and 11 Beta-Hydroxylase Deficiency in a Child Carrying a Homozygous Deletion Hybrid CYP11B2 (Aldosterone Synthase)-CYP11B1 (11 Beta-Hydroxylase). Clin Genet (2004) 66(3):229–35. doi: 10.1111/j.1399-0004.2004.00291.x 15324322

[46] Turan I Tastan M Boga DD Gurbuz F Kotan LD Tuli A. 21-Hydroxylase Deficiency: Mutational Spectrum and Genotype-Phenotype Relations Analyses by Next-Generation Sequencing and Multiplex Ligation-Dependent Probe Amplification. Eur J Med Genet (2020) 63(4):103782. doi: 10.1016/j.ejmg.2019.103782 31586465

[47] Gangodkar P Khadilkar V Raghupathy P Kumar R Dayal AA Dayal D. Clinical Application of a Novel Next Generation Sequencing Assay for CYP21A2 Gene in 310 Cases of 21-Hydroxylase Congenital Adrenal Hyperplasia From India. Endocrine (2021) 71(1):189–98. doi: 10.1007/s12020-020-02494-z 32948948

[48] Stephens Z Milosevic D Kipp B Grebe S Iyer RK Kocher JP. PB-Motif-A Method for Identifying Gene/Pseudogene Rearrangements With Long Reads: An Application to CYP21A2 Genotyping. Front Genet (2021) 12:716586. doi: 10.3389/fgene.2021.716586 34394200 PMC8355628

[49] Wang W Han R Yang Z Zheng S Li H Wan Z. Targeted Gene Panel Sequencing for Molecular Diagnosis of Congenital Adrenal Hyperplasia. J Steroid Biochem Mol Biol (2021) 211:105899. doi: 10.1016/j.jsbmb.2021.105899 33864926

[50] Mohammadi MM Bavi O. DNA Sequencing: An Overview of Solid-State and Biological Nanopore-Based Methods. Biophys Rev (2021) 14:99–110. doi: 10.1007/s12551-021-00857-y PMC8609259 34840616

[51] Goto Y Akahori R Yanagi I Takeda KI. Solid-State Nanopores Towards Single-Molecule DNA Sequencing. J Hum Genet (2020) 65(1):69–77. doi: 10.1038/s10038-019-0655-8 31420594

[52] Girgis H DuPai CD Lund J Reeder J Guillory J Durinck S. Single-Molecule Nanopore Sequencing Reveals Extreme Target Copy Number Heterogeneity in Arylomycin-Resistant Mutants. Proc Natl Acad Sci USA (2021) 118(1):e2021958118. doi: 10.1073/pnas.2021958118 33443214 PMC7817135

[53] Owerbach D Crawford YM Draznin MB. Direct Analysis of CYP21B Genes in 21-Hydroxylase Deficiency Using Polymerase Chain Reaction Amplification. Mol Endocrinol (1990) 4(1):125–31. doi: 10.1210/mend-4-1-125 2325662

[54] Ezquieta B Oliver A Gracia R Gancedo PG. Analysis Ofsteroid 21-Hydroxylase Mutations in the Spanish Population. HumGenet (1995) 96:198–204. doi: 10.1007/BF00207379 7635470

[55] Ezquieta B Varela JM Jariego C Oliver A Gracia R. Nonisotopic Detection of Point Mutations in CYP21B Gene in Steroid21-Hydroxylase Deficiency. Clin Chem (1996) 42:1108–10. doi: 10.1093/clinchem/42.7.1108 8674198

[56] Ezquieta B Cueva E Varela J Oliver A Fernández J Jariego C. Nonclassical 21-Hydroxylase Deficiency in Children: Association of ACTH-Stimulated 17OH Progesterone With Risk for Compound Heterozygosity for Severe Mutations. Acta Paediatr (2002) 91:892–8. doi: 10.1080/080352502760148595 12222711

[57] Concolino P Mello E Patrosso MC Penco S Zuppi C Capoluongo E. P.H282N and P.Y191H: 2 Novel CYP21A2 Mutations in Italian Congenital Adrenal Hyperplasia Patients. Metabolism (2012) 61(4):519–24. doi: 10.1016/j.metabol.2011.08.008 22014889

[58] Brønstad I Breivik L Methlie P Wolff AS Bratland E Nermoen I. Functional Studies of Novel CYP21A2 Mutations Detected in Norwegian Patients With Congenital Adrenal Hyperplasia. Endocr Connect (2014) 3(2):67–74. doi: 10.1530/EC-14-0032 24671123 PMC3987286

[59] Karlsson L de Paula D Gori AL D'Almeida C Östberg LJ Persson B. Novel Non-Classic CYP21A2 Variants, Including Combined Alleles, Identified in Patients With Congenital Adrenal Hyperplasia. Clin Biochem (2019) 73:50–6. doi: 10.1016/j.clinbiochem.2019.07.009 31344365

[60] Robins T Carlsson J Sunnerhagen M Wedell A Persson B. Molecular Model of Human CYP21 Based on Mammalian CYP2C5: Structural Features Correlate With Clinical Severity of Mutations Causing Congenital Adrenal Hyperplasia. Mol Endocrinol (2006) 20(11):2946–64. doi: 10.1210/me.2006-0172 16788163

[61] Haider S Islam B D'Atri V Sgobba M Poojari C Sun L. Structure-Phenotype Correlations of Human CYP21A2 Mutations in Congenital Adrenal Hyperplasia. Proc Natl Acad Sci USA (2013) 110(7):2605–10. doi: 10.1073/pnas.1221133110 PMC3574933 23359706

[62] Pallan PS Lei L Wang C Waterman WR Guengerich FP Egli M. Research Resource: Correlating Human Cytochrome P450 21a2 Crystal Structure and Phenotypes of Mutations in Congenital Adrenal Hyperplasia. Mol Endocrinol (2015) 29(9):1375–84. doi: 10.1210/ME.2015-1127 PMC4552440 26172259

[63] de Carvalho DF Miranda MC Gomes LG Madureira G Marcondes JA Billerbeck AE. Molecular CYP21A2 Diagnosis in 480 Brazilian Patients With Congenital Adrenal Hyperplasia Before Newborn Screening Introduction. Eur J Endocrinol (2016) 175(2):107–16. doi: 10.1530/EJE-16-0171 27185867

[64] Xu Z Chen W Merke DP McDonnell NB. Comprehensive Mutation Analysis of the CYP21A2 Gene. An Efficient Multistep Approach to the Molecular Diagnosis of Congenital Adrenal Hyperplasia. J Mol Diagn (2013) 15(6):745–53. doi: 10.1016/j.jmoldx.2013.06.001 PMC5803549 24071710

[65] Concolino P. Issues With the Detection of Large Genomic Rearrangements in Molecular Diagnosis of 21-Hydroxylase Deficiency. Mol Diagn Ther (2019) 23(5):563–7. doi: 10.1007/s40291-019-00415-z 31317337

[66] Tardy V Menassa R Sulmont V Lienhardt-Roussie A Lecointre C Brauner R. Phenotype-Genotype Correlations of 13 Rare CYP21A2 Mutations Detected in 46 Patients Affected With 21-Hydroxylase Deficiency and in One Carrier. J Clin Endocrinol Metab (2010) 95(3):1288–300. doi: 10.1210/jc.2009-1202 20080860

[67] Araujo RS Billerbeck AE Madureira G Mendonca BB Bachega TA. Substitutions in the CYP21A2 Promoter Explain the Simple-Virilizing Form of 21-Hydroxylase Deficiency in Patients Harbouring a P30L Mutation. Case Rep Clin Endocrinol (Oxf) (2005) 62(2):132–6. doi: 10.1111/j.1365-2265.2005.02184.x 15670187

[68] Friães A Rêgo AT Aragüés JM Moura LF Mirante A Mascarenhas MR. CYP21A2 Mutations in Portuguese Patients With Congenital Adrenal Hyperplasia: Identification of Two Novel Mutations and Characterization of Four Different Partial Gene Conversions. Mol Genet Metab (2006) 88(1):58–65. doi: 10.1016/j.ymgme.2005.11.015 16427797

[69] Parajes S Quinteiro C Domínguez F Loidi L. High Frequency of Copy Number Variations and Sequence Variants at CYP21A2 Locus: Implication for the Genetic Diagnosis of 21-Hydroxylase Deficiency. PloS One (2008) 3(5):e2138. doi: 10.1371/journal.pone.0002138 18478071 PMC2364643

[70] Wedell A Stengler B Luthman H. Characterization of Mutations on the Rare Duplicated C4/CYP21 Haplotype in Steroid 21-Hydroxylase Deficiency. Hum Genet (1994) 94(1):50–4. doi: 10.1007/BF02272841 8034294

[71] Cantürk C Baade U Salazar R Storm N Pörtner R Höppner W. Sequence Analysis of CYP21A1P in a German Population to Aid in the Molecular Biological Diagnosis of Congenital Adrenal Hyperplasia. Clin Chem (2011) 57(3):511–7. doi: 10.1373/clinchem.2010.156893 21148302

[72] Ezquieta B Muñoz-Pacheco R Santomé L Ferreiro B García D Casado C. Pitfalls in the Molecular Diagnosis of 21OH Deficiency Due to Point Mutations Identification Without Further Characterizations of Gene Deletions and Duplications. Horm Res (2008) 71(Suppl 1):45. 47th Annual Meeting ESPE. Horm Res. 2008. 70, pp. 45 - 45. 01/01/2008. ISSN. doi: 10.1159/000157521 19039236

[73] Schulze E Bettendorf M Maser-Gluth C Decker M Schwabe U. Allele-Dropout Using PCR-Based Diagnosis for the Splicing Mutation in Intron-2 of the CYP21B-Gene: Successful Amplification With a Taq/Pwo-Polymerase Mixture. Endocr Res (1998) 24(3-4):637–41. doi: 10.3109/07435809809032662 9888552

[74] Ezquieta B Jariego C Varela JM Oliver A Gracia R. Microsatellite Markers in the Indirect Analysis of the Steroid 21-Hydroxylase Gene. Prenat Diagn (1997) 17:429–34. doi: 10.1002/(sici)1097-0223(199705)17:5<429::aid-pd77>3.0.co;2-9 9178317

[75] Coates BS Sumerford DV Miller NJ Kim KS Sappington TW Siegfried BD. Comparative Performance of Single Nucleotide Polymorphism and Microsatellite Markers for Population Genetic Analysis. J Hered (2009) 100(5):556–64. doi: 10.1093/jhered/esp028 19525239

[76] New MI Tong YK Yuen T Jiang P Pina C Chan KC. Noninvasive Prenatal Diagnosis of Congenital Adrenal Hyperplasia Using Cell-Free Fetal DNA in Maternal Plasma. J Clin Endocrinol Metab (2014) 99(6):E1022–30. doi: 10.1210/jc.2014-1118 PMC4037720 24606108

[77] Fitness J Dixit N Webster D Torresani T Pergolizzi R Speiser PW. Genotyping of CYP21, Linked Chromosome 6p Markers, and a Sex-Specific Gene in Neonatal Screening for Congenital Adrenal Hyperplasia. J Clin Endocrinol Metab (1999) 84(3):960–6. doi: 10.1210/jcem.84.3.5550 10084579

[78] Karell K Klinger N Holopainen P Levo A Partanen J. Major Histocompatibility Complex (MHC)-Linked Microsatellite Markers in a Founder Population. Tissue Antigens (2000) 56(1):45–51. doi: 10.1034/j.1399-0039.2000.560106.x 10958355

[79] Soriano L Velázquez M Ezquieta B. Usefulness of Molecular Analysis in the Differential Diagnosis of Congenital 21-Hidroxylase Deficiency Detected in Neonatal Screening. Med Clin (Barc) (2011) 136(7):313–4. doi: 10.1016/j.medcli.2009.06.008 19766262

[80] Dulín E Ezquieta B. Newborn Screening of Congenital Adrenal Hyperplasia. Endocrinol Diabetes Nutr (2018) 65:1–4. doi: 10.1016/j.endien.2017.11.015 29241677

[81] Balcells C Gili T Pérez J Corripio R. Pseudohypoaldosteronism Without Nephropathy Masking Salt-Wasting Congenital Adrenal Hyperplasia Genetically Confirmed. BMJ Case Rep (2013) 2013:bcr2012008281. doi: 10.1136/bcr-2012-008281 PMC3603811 23370958

[82] Tuhan HU Catli G Anik A Onay H Dundar B Bober E. Cross-Reactivity of Adrenal Steroids With Aldosterone may Prevent the Accurate Diagnosis of Congenital Adrenal Hyperplasia. J Pediatr Endocrinol Metab (2015) 28(5-6):701–4. doi: 10.1515/jpem-2014-0170 25503463

[83] Nordenström A Thilén A Hagenfeldt L Larsson A Wedell A. Genotyping is a Valuable Diagnostic Complement to Neonatal Screening for Congenital Adrenal Hyperplasia Due to Steroid 21-Hydroxylase Deficiency. J Clin Endocrinol Metab (1999) 84(5):1505–9. doi: 10.1210/jcem.84.5.5651 10323369

[84] Gidlöf S Falhammar H Thilén A von Döbeln U Ritzén M Wedell A. One Hundred Years of Congenital Adrenal Hyperplasia in Sweden: A Retrospective, Population-Based Cohort Study. Lancet Diabetes Endocrinol (2013) 1(1):35–42. doi: 10.1016/S2213-8587(13)70007-X 24622265

[85] Falhammar H Wedell A Nordenström A. Biochemical and Genetic Diagnosis of 21-Hydroxylase Deficiency. Endocrine (2015) 50(2):306–14. doi: 10.1007/s12020-015-0731-6 26336836

[86] Marino S Perez N Ramírez P Pujana M Dratler G Belgorosky A. Molecular Analysis of the CYP21A2 Gene in Dried Blood Spot Samples. Medicina (B Aires) (2020) 80(3):197–202. 32442933

[87] Castro PS Rassi TO Araujo RF Pezzuti IL Rodrigues AS Bachega TA. High Frequency of non-Classical Congenital Adrenal Hyperplasia Form Among Children With Persistently Elevated Levels of 17-Hydroxyprogesterone After Newborn Screening. J Pediatr Endocrinol Metab (2019) 32(5):499–504. doi: 10.1515/jpem-2018-0398 31028712

[88] New M. Inborn Errors of Adrenal Steroidogenesis. Mol Cell Endocrinol (2003) 211(1-2):75–83. doi: 10.1016/j.mce.2003.09.013 14656479

[89] Ezquieta B Fernandez ML Dulín E Rodriguez D Rodríguez A. Prevalence of Frequent Recessive Diseases in the Spanish Population Through DNA Analyses on Samples From the Neonatal Screening. Med Clin (Barc) (2005) 125(13):493–5. doi: 10.1157/13080213 16238926

[90] Admoni O Israel S Lavi I Gur M Tenenbaum-Rakover Y. Hyperandrogenism in Carriers of CYP21 Mutations: The Role of Genotype. Clin Endocrinol (Oxf) (2006) 64(6):645–51. doi: 10.1111/j.1365-2265.2006.02521.x 16712666

[91] Ezquieta B Oyarzabal M Barrio R Luzuriaga C Hermoso F Lechuga JL. Monogenic and Polygenic Models Detected in Steroid 21-Hydroxylase Deficiency-Related Paediatric Hyperandrogenism. Horm Res (2009) 71(1):28–37. doi: 10.1159/000173739 19039234

[92] Guarnotta V Niceta M Bono M Marchese S Fabiano C Indelicato S. Clinical and Hormonal Characteristics in Heterozygote Carriers of Congenital Adrenal Hyperplasia. J Steroid Biochem Mol Biol (2020) 198:105554. doi: 10.1016/j.jsbmb.2019.105554 31805392

[93] Turcu AF Rege J Auchus RJ Rainey WE. 11-Oxygenated Androgens in Health and Disease. Nat Rev Endocrinol (2020) 16(5):284–96. doi: 10.1038/s41574-020-0336-x PMC7881526 32203405

[94] Sumińska M Bogusz-Górna K Wegner D Fichna M. Non-Classic Disorder of Adrenal Steroidogenesis and Clinical Dilemmas in 21-Hydroxylase Deficiency Combined With Backdoor Androgen Pathway. Mini-Review Case Rep Int J Mol Sci (2020) 21(13):4622. doi: 10.3390/ijms21134622 PMC7369945 32610579

[95] Costa-Barbosa FA Carvalho VM Oliveira KC Vieira JG Kater CE. Reassessment of Predictive Values of ACTH-Stimulated Serum 21-Deoxycortisol and 17-Hydroxyprogesterone to Identify CYP21A2 Heterozygote Carriers and Nonclassic Subjects. Clin Endocrinol (Oxf) (2021) 95(4):677–85. doi: 10.1111/cen.14550 34231242

[96] Speiser PW Dupont B Rubinstein P Piazza A Kastelan A New MI. High Frequency of Nonclassical Steroid 21-Hydroxylase Deficiency. Am J Hum Genet (1985) 37:650–67. PMC1684620 9556656

[97] Pignatelli D Carvalho BL Palmeiro A Barros A Guerreiro SG Macut D. The Complexities in Genotyping of Congenital Adrenal Hyperplasia: 21-Hydroxylase Deficiency. Front Endocrinol (Lausanne) (2019) 10:432. doi: 10.3389/fendo.2019.00432 31333583 PMC6620563

[98] Livadas S Bothou C. Management of the Female With Non-Classical Congenital Adrenal Hyperplasia (NCCAH): A Patient-Oriented Approach. Front Endocrinol (Lausanne) (2019) 10:366. doi: 10.3389/fendo.2019.00366 31244776 PMC6563652

[99] Trakakis E Loghis C Kassanos D. Congenital Adrenal Hyperplasia Because of 21-Hydroxylase Deficiency. A Genetic Disorder of Interest to Obstetricians and Gynecologists. Obstet Gynecol Surv (2009) 64(3):177–89. doi: 10.1097/OGX.0b013e318193301b 19228439

[100] Chatziaggelou A Sakkas EG Votino R Papagianni M Mastorakos G. Assisted Reproduction in Congenital Adrenal Hyperplasia. Front Endocrinol (Lausanne) (2019) 10:723. doi: 10.3389/fendo.2019.00723 31708872 PMC6819309

[101] Narasimhan ML Khattab A. Genetics of Congenital Adrenal Hyperplasia and Genotype-Phenotype Correlation. Fertil Steril (2019) 111(1):24–9. doi: 10.1016/j.fertnstert.2018.11.007 30611409

[102] Claahsen-van HL Speiser PW Ahmed SF Arlt W Auchus RJ Falhammar H. Congenital Adrenal Hyperplasia - Current Insights in Pathophysiology, Diagnostics and Management. Endocr Rev (2022) 43(1):91–159. doi: 10.1210/endrev/bnab016 33961029 PMC8755999

