# Supplementary material for: Molecular Diagnosis of Steroid 21-Hydroxylase Deficiency: A Practical Approach
Source: Front Endocrinol (Lausanne). 2022 Mar 29;13:834549. doi: 10.3389/fendo.2022.834549 (PMC9001848; doi:10.3389/fendo.2022.834549)
Supplement: Supplementary file 1 [file Table_1.docx]

| **Supplementary Table 1.** Distribution of frequencies of 21-OHD alleles in fully genotyped patients with confirmed severe clinical deficiency, and in clinically unaffected individuals from the general population (partners of carriers or patients with severe *CYP21A2* alleles). All patients were characterized between 1995 and 2009. [*Adapted from Ezquieta et al 2010*]. | | | | | |
| --- | --- | --- | --- | --- | --- |
| 21OHD alleles^1^ | | Patients^2^ | | Partners of couples of patients or carriers (general population) | |
|  |  |  |  |  |  |
| **SEVERE** | MILD or *NORMAL* | n=255 (510 alleles) | | n=230 | |
|  |  | % (Alleles) | 95% confidence interval | Alleles (Allele frequency) | **Carrier frequency %** (95%CI) |
| **Gene deletions** |  | 14.5 (74) | 1.4-17.7 | 0/460 |  |
| **Gene conversions^3^** |  | 12 (61) | 9.0-14.9 | 1^3^* (0.002) | **0.4** (0.01-2.4) |
| **Severe double mutations^4^** |  | 9.8 (50) | 7.1-12.5 | 1 (0.002) | **0.4** (0.01-2.4) |
| **p.Pro30Leu(5´Conv)^5,6^** |  | 1.7 (9^6^) | 0.5-3.0 | 0/460 |  |
|  | p.Pro30Leu^5^ | 0.4 (2^6^) | 0.1-1.4 |  |  |
| **IVS2-13A/C>G** |  | 28 (142) | 23.9-31.8 | 1 (0.002) | **0.4** (0.01-2.4) |
| **8nt deletion exon 3** |  | 2 (11) | 0.8-3.5 | 0/460 |  |
| **p.Ile172Asn^6^** |  | 5.7 (29^6^) | 3.6-7.8 | 0/460 |  |
| **p.[Ile236Asn;Val237Glu;Met239Lys]** |  | 1.2 (6) | 0.1-2.2 | 0/460 |  |
| **p.Val281Leu; IVS2+5G>A^7^** |  | 1.5 (8) | 0.4-2.7 | 0/460 |  |
|  | p.Val281Leu^6^ | 0.6 (3^6^) | 0.1-1.7 | 27 (0.060) | **11.7** (7.4-16.1) |
| **Phe306fs** |  | 1.5 (8) | 0.4-2.7 | 0/460 |  |
| **p.Gln318Stop** |  | 12 (62) | 9.2-15.1 | 1 (0.002) | **0.4** (0.01-2.4) |
|  | ***Stop318Dup*^8^** | 0 | 0^8^* | *6 (0.013)* | ***2.6*** *(0.3-4.9)* |
| **p.Arg356Trp** |  | 4 (21) | 2.3-5.9 | 0/460 |  |
| **p.Arg426His^6^** |  | 1 (5^6^) | 0.3-2.2) | 0/460 |  |
|  | p.Pro453Ser | 0 | 0 | 2 (0.004) | **0.8** (0.1-3.1) |
| Panel coverage^9^(alleles) | | 491/510 (96.3%) | 94.5-98.0 |  |  |
| Capacity of the panel to completely characterise patients (two segregated mutant alleles) | | 241/255 (94.5%) | 91.5-97.5 |  | |
| Patients partially characterised by the panel (one mutant allele)^8^ | | 9/255 (3.5%) | 1,1-5,9 |  | |
| D6S273 homozygosity (rare alleles)^9^ | | 5/255 (2%) | 0.6-4.5 |  | |
| **Carrier frequency for severe 21-OHD alleles** |  |  |  |  | **1.7** (0.5-4.4)  **1:58** |
| **Carrier frequency for mild 21-OHD alleles** |  |  |  |  | **12.6** (8.1-17.1)  **1:8** |
| ^1^ Point variants are listed following their sequential order in the gene. The traditional names are used. For a nomenclature according HGVS recommendations refer to the legend in Figure 1. | | | | | |
| ^2^ Only fully characterized, non-related patients for whom segregation analysis results were available are included. | | | | | |
| ^3^ Both PCR-unamplifiable alleles (large gene conversions detected by Southern blotting) and PCR-amplifiable alleles including point variants in several exons. *Showing the mentioned Southern pattern and hemizygosity for all the polymorphic nucleotides upon DNA sequencing. | | | | | |
| ^4^ Two common point variants in a single allele, at least one severe. | | | | | |
| ^5^ p.Pro30Leu alleles including the 5´conversion in the promoter region (accession number CP015779, Cooper et al 2008). | | | | | |
| ^6^ Pathological alleles predominantly (44/48) associated with severe virilizing forms without SW. | | |  |  |  |
| ^7^ 8/9 severe alleles in SW forms apparently carrying the mild (isolated) point variant p.Val281Leu in compound heterozygosity with classical alterations.  ^8^ p.Gln318Stop in gene duplicated alleles is a non-deficient allele (Koppens et al., 2002; Ezquieta et al., 2006). * One patient with an SV form with p.Ile172Asn showed the Stop318Dup in his second allele. He was not included in this series as he was considered a partially characterized patient. | | | | | |
| ^9^ Uncommon allele variants carrying point variants previously documented in other populations or first described in Spanish patients (Ezquieta et al, 1999; Lobato et al, 1999; Ezquieta et al, 2002a;Cooper et al, 2008 ) completed the characterization: p.[Ile236Lys;Val237Glu;Met239Lys], p.[Gln316Stop;Val281Leu], p.Leu65fs, p.Gly291Ser, p.G424S in homozygosity, microsatellite marker homozygosity revealing unknown consanguinity (Ezquieta et al, 1999) p.Arg483fs (2671insCdelGG) in two patients, p.Trp19Stop, p.Thr348fs in hemizygogity due to gene deletions or conversions, and p.Phe306fsdelT, p.Gln228Stop, p.Gln474Stop, p.Gln153Stop, p.Thr348fs in compound heterozygous patients with a common mutation in their second alleles. | | | | | |
|  | | | | | |

Ezquieta B, Oyarzábal M, Jariego CM, JM Varela, M Chueca. A novel frameshift in the first exon of the 21-OH gene found in homozygosity in an apparently nonconsanguineous family. Horm Res. 1999;51(3):135–141. doi: 10.1159/000023346.

Lobato MN, Ordó˜nez-Sánchez ML, Tusié-Luna MT, Meseguer A. Mutation analysis in patients with congenital adrenal hyperplasia in the Spanish population: identification of putative novel steroid 21-hydroxylase deficiency alleles associated with the classic form of the disease. Hum Hered. 1999Jun;49(3):169–175. doi: 10.1159/000022866

Cooper DN, Ball EV, Stenson PD, Krawczak M. 2008. The Human Gene Mutation Database (HGMD). Available at: http://www.hgmd.cf.ac. uk/ac/index.php [10 May 2009].
