# Supplementary material for: Molecular Diagnosis of Steroid 21-Hydroxylase Deficiency: A Practical Approach
Source: Front Endocrinol (Lausanne). 2022 Mar 29;13:834549. doi: 10.3389/fendo.2022.834549 (PMC9001848; doi:10.3389/fendo.2022.834549)
Supplement: Supplementary file 2 [file Table_2.docx]

| **Supplementary Table 2.** Distribution of frequencies of the common 21OH-deficient alleles in paediatric patients (groups I [NCF] and II [hyperandrogenic patients with 17-OHP after ACTH below the threshold for NCF]) and comparison with general population. [*Adapted from Ezquieta et al 2009*]. | | | | | | | |
| --- | --- | --- | --- | --- | --- | --- | --- |
|  |  |  |  |  |  |  |  |
|  | Paediatric patients (n=681) | | | | Carriers in general population^c^ (n=230) | | p-value |
|  | Group I  (n=375, 750 alleles) Female=290/Male=85 | | Group II  (n=306) Female=269/Male=37 | |  |  | Carrier frequency Group II vs general population^c^ |
|  | Alleles (n) | Allele frequency | Alleles (n) | Allele frequency | Alleles (n) | Allele frequency |  |
| Deletions^a^ | 27 | 3.6% | 6 | 1% | 0 | 0 |  |
| Conversions | 17 | 2.3% | 6 | 1% | 1 | 0.4% |  |
| 655G | 34 | 4.5% | 6 | 1% | 1 | 0.4% |  |
| Del8 | 2 | 0.3% | 0 | 0 | 0 | 0 |  |
| I172N | 27 | 3.6% | 2 | 0.3% | 0 | 0 |  |
| Ex6A | 1 | 0.1% | 0 | 0 | 0 | 0 |  |
| 306insT | 2 | 0.3% | 0 | 0 | 0 | 0 |  |
| Q318X | 18 | 2.4% | 7 | 1.1% | 1 | 0.4% |  |
| R356W | 8 | 1.1% | 2 | 0.3% | 0 | 0 |  |
| Double sev mutation | 16 | 2.1% | 1 | 0.2% | 1 | 0.4% |  |
|  |  |  |  |  |  |  |  |
| P30L^b^ | 18 | 2.4% | 4 | 0.7% | 0 | 0 |  |
| V281L | 344 | 45.9% | 102 | 16.7% | 27 | 11.7% |  |
| P453S | 32 | 4.0 | 5 | 0.8% | 2 | 0.8% |  |
|  |  | |  | |  | |  |
| Alleles^d^ | n | %^d^ | n | % | n | % |  |
| SEVERE | 152 | 41% | 30 | 10% | 4 | 1.6% | **0.003** |
| MILD |  |  | 111 | 36% | 29 | 12.6% | **0.0002** |
|  |  |  |  |  |  |  |  |
| ^a^ Severe and ^b^mild variants, upper and lower parts of the Table. Point pathological variants are included following the order of the exons in which they are located. For a nomenclature according HGVS recommendations refer to the legend in Figure 1. | | | | | | | |
| ^c^ Carriers from the general population (group of couples analysed for genetic counseling, Ezquieta 2010, see Supplementary Table 1). | | | | | | | |
| ^d^ The number (n) of severe or mild alleles in each group of patients, their family members, and in general population, is indicated. Patients who were compound heterozygous for a severe variant are indicated in Group I. Only severe alleles are scored in this biallelic group. | | | | | | | |
