# Supplementary material for: Molecular Diagnosis of Steroid 21-Hydroxylase Deficiency: A Practical Approach
Source: Front Endocrinol (Lausanne). 2022 Mar 29;13:834549. doi: 10.3389/fendo.2022.834549 (PMC9001848; doi:10.3389/fendo.2022.834549)
Supplement: Supplementary file 3 [file Table_3.docx]

| **Supplementary Table 3.** Levels of 17-OHP, basal and post-ACTH in genotyped patients with NCF of the deficiency. [*Adapted from Alonso & Ezquieta 2012*]. | | | | | |
| --- | --- | --- | --- | --- | --- |
| Genotype | Patients^a^ | 17-OHP (ng/mL) | | | |
|  |  | basal^b,d^ | Confidenze interval (95%) | postACTH^b^ | Confidenze interval (95%) |
| [p.Val282Leu];[p.Val282Leu] | 192/122 | 14.3 (12.3) | 12.5-16.0 | 46.1 (27.4) | 41.2-51.0 |
| [p.Val282Leu];[p.Pro454Ser] | 19/19 | 10.2 (8.0) | 6,3-14,1 | 25,5 (9,1) | 21,1-29,9 |
| [p.Val282Leu];Gene deletion | 51/28 | 24.0 (18.2) | 18.6-29.4 | 53.3 (33.9) | 39.9-66.1 |
| [p.Val282Leu];Gene conversion |  |  |  |  |  |
| [p.Val282Leu];[c.293-13C>G] | 28/16 | 29.7 (35.0) | 16.1-43.3 | 62.2 (42.0) | 39.8-84.6 |
| [p.Val282Leu];[p.Gln319*]^c^ | 20/13 | 36.9 (24,8) | 25.3-48.5 | 79.9 (37.1) | 57.5-102.3 |
| Mild/mild | 211/141 | 13.9 (12.0) | 12.3-15.5 | 43.3 (26.6) | 38.9-47.7 |
| Severe/mild | 99/57 | 27.3 (24.8) | 22.3-32.3 | 61.9 (37.9) | 51.8-71.9 |
|  |  |  |  |  |  |
| ^a^Number of patients with available 17-OHP data. Levels reported as higher than (>) are not scored. There were other genotypes not included in this Table due to the small number of patients (<20). Compound heterozygosity [p.Val282Leu];[p.Pro454Ser] has been included because of the lower values observed in this genotype. | | | | | |
| ^b^Basal and ACTH-stimulated 17-OHP: media (standard deviation). | | | | | |
| ^c^Only those patients carrying the severe allele (gene duplications excluded). | | | | | |
| ^d^Only 3/484 with basal levels of 17-OHP below 1ng/mL, and 5/484 below 2ng/mL.  For a nomenclature according HGVS recommendations refer to the legend in Figure 1. | | | | | |
|  | | | | | |
